# Supplementary material for: Modeling glioblastoma heterogeneity as a dynamic network of cell states
Source: Mol Syst Biol. 2021 Sep 16;17(9):e10105. doi: 10.15252/msb.202010105 (PMC8444284; doi:10.15252/msb.202010105)
Supplement: Supplementary file 6 — Source Data for Figure 5 [file MSB-17-e10105-s004.zip › Figure5A_sourcedata/GSEA_3071/hallmarks_stateI.GseaPreranked.1623742531165/HALLMARK_MITOTIC_SPINDLE.html]

Details for gene set HALLMARK\_MITOTIC\_SPINDLE[GSEA]

|  || Dataset | state13071 |
| Phenotype | NoPhenotypeAvailable |
| Upregulated in class | na\_neg |
| GeneSet | HALLMARK\_MITOTIC\_SPINDLE |
| Enrichment Score (ES) | -0.19154988 |
| Normalized Enrichment Score (NES) | -0.96986395 |
| Nominal p-value | 0.5056818 |
| FDR q-value | 0.57920116 |
| FWER p-Value | 1.0 |
Table: GSEA Results Summary

  

Fig 1: Enrichment plot: HALLMARK\_MITOTIC\_SPINDLE      
 Profile of the Running ES Score & Positions of GeneSet Members on the Rank Ordered List

  

| PROBE | GENE SYMBOL | GENE\_TITLE | RANK IN GENE LIST | RANK METRIC SCORE | RUNNING ES | CORE ENRICHMENT || 1 | STK38L |  |  | 39 | 1.441 | 0.0226 | No |
| 2 | BIN1 |  |  | 127 | 0.967 | 0.0199 | No |
| 3 | SORBS2 |  |  | 156 | 0.905 | 0.0330 | No |
| 4 | VCL |  |  | 162 | 0.891 | 0.0526 | No |
| 5 | GSN |  |  | 265 | 0.720 | 0.0397 | No |
| 6 | TIAM1 |  |  | 341 | 0.636 | 0.0328 | No |
| 7 | PALLD |  |  | 342 | 0.636 | 0.0478 | No |
| 8 | FLNA |  |  | 373 | 0.614 | 0.0535 | No |
| 9 | AKAP13 |  |  | 375 | 0.611 | 0.0676 | No |
| 10 | NOTCH2 |  |  | 446 | 0.551 | 0.0601 | No |
| 11 | ARHGAP29 |  |  | 508 | 0.502 | 0.0541 | No |
| 12 | EPB41 |  |  | 536 | 0.488 | 0.0577 | No |
| 13 | NEDD9 |  |  | 538 | 0.487 | 0.0689 | No |
| 14 | NF1 |  |  | 617 | 0.445 | 0.0566 | No |
| 15 | TUBA4A |  |  | 664 | 0.425 | 0.0532 | No |
| 16 | MID1IP1 |  |  | 688 | 0.418 | 0.0563 | No |
| 17 | MYH9 |  |  | 701 | 0.411 | 0.0625 | No |
| 18 | PKD2 |  |  | 754 | 0.396 | 0.0566 | No |
| 19 | DST |  |  | 804 | 0.380 | 0.0513 | No |
| 20 | RICTOR |  |  | 809 | 0.378 | 0.0590 | No |
| 21 | FGD6 |  |  | 855 | 0.364 | 0.0544 | No |
| 22 | RASA1 |  |  | 935 | 0.342 | 0.0394 | No |
| 23 | CDC42 |  |  | 1056 | 0.316 | 0.0118 | No |
| 24 | EPB41L2 |  |  | 1170 | 0.295 | -0.0142 | No |
| 25 | ABI1 |  |  | 1200 | 0.288 | -0.0159 | No |
| 26 | WASF2 |  |  | 1207 | 0.286 | -0.0109 | No |
| 27 | SHROOM1 |  |  | 1212 | 0.286 | -0.0053 | No |
| 28 | RAB3GAP1 |  |  | 1257 | 0.275 | -0.0117 | No |
| 29 | ARHGEF2 |  |  | 1347 | 0.260 | -0.0316 | No |
| 30 | PCGF5 |  |  | 1436 | -0.252 | -0.0513 | No |
| 31 | CYTH2 |  |  | 1443 | -0.252 | -0.0471 | No |
| 32 | FARP1 |  |  | 1496 | -0.257 | -0.0563 | No |
| 33 | FLNB |  |  | 1591 | -0.267 | -0.0775 | No |
| 34 | TTK |  |  | 1620 | -0.270 | -0.0793 | No |
| 35 | SSH2 |  |  | 1637 | -0.272 | -0.0775 | No |
| 36 | SMC3 |  |  | 1650 | -0.274 | -0.0746 | No |
| 37 | MYH10 |  |  | 1670 | -0.276 | -0.0736 | No |
| 38 | KIF2C |  |  | 1735 | -0.284 | -0.0856 | No |
| 39 | CCNB2 |  |  | 1757 | -0.288 | -0.0850 | No |
| 40 | CENPJ |  |  | 1768 | -0.290 | -0.0811 | No |
| 41 | TBCD |  |  | 1796 | -0.293 | -0.0821 | No |
| 42 | CEP250 |  |  | 1861 | -0.300 | -0.0937 | No |
| 43 | INCENP |  |  | 2018 | -0.320 | -0.1317 | No |
| 44 | ARHGDIA |  |  | 2122 | -0.338 | -0.1538 | No |
| 45 | EZR |  |  | 2123 | -0.338 | -0.1458 | No |
| 46 | PLK1 |  |  | 2206 | -0.353 | -0.1615 | No |
| 47 | SHROOM2 |  |  | 2230 | -0.360 | -0.1597 | No |
| 48 | SMC1A |  |  | 2240 | -0.361 | -0.1538 | No |
| 49 | NET1 |  |  | 2290 | -0.370 | -0.1594 | No |
| 50 | MYO9B |  |  | 2401 | -0.393 | -0.1823 | Yes |
| 51 | BUB1 |  |  | 2416 | -0.396 | -0.1771 | Yes |
| 52 | NIN |  |  | 2447 | -0.401 | -0.1764 | Yes |
| 53 | YWHAE |  |  | 2453 | -0.402 | -0.1684 | Yes |
| 54 | AURKA |  |  | 2467 | -0.404 | -0.1627 | Yes |
| 55 | KIF11 |  |  | 2488 | -0.410 | -0.1589 | Yes |
| 56 | RACGAP1 |  |  | 2522 | -0.419 | -0.1586 | Yes |
| 57 | APC |  |  | 2550 | -0.427 | -0.1565 | Yes |
| 58 | PCM1 |  |  | 2555 | -0.428 | -0.1476 | Yes |
| 59 | CDK5RAP2 |  |  | 2564 | -0.431 | -0.1397 | Yes |
| 60 | FSCN1 |  |  | 2569 | -0.432 | -0.1307 | Yes |
| 61 | PPP4R2 |  |  | 2586 | -0.436 | -0.1252 | Yes |
| 62 | KNTC1 |  |  | 2613 | -0.443 | -0.1223 | Yes |
| 63 | KIF22 |  |  | 2683 | -0.468 | -0.1314 | Yes |
| 64 | ANLN |  |  | 2702 | -0.472 | -0.1256 | Yes |
| 65 | PRC1 |  |  | 2705 | -0.472 | -0.1151 | Yes |
| 66 | NDC80 |  |  | 2723 | -0.478 | -0.1088 | Yes |
| 67 | CENPF |  |  | 2761 | -0.490 | -0.1080 | Yes |
| 68 | BRCA2 |  |  | 2772 | -0.495 | -0.0993 | Yes |
| 69 | TOP2A |  |  | 2842 | -0.517 | -0.1073 | Yes |
| 70 | ECT2 |  |  | 2858 | -0.523 | -0.0993 | Yes |
| 71 | KIF4A |  |  | 2953 | -0.574 | -0.1132 | Yes |
| 72 | KIF23 |  |  | 2961 | -0.579 | -0.1017 | Yes |
| 73 | PREX1 |  |  | 3005 | -0.605 | -0.1000 | Yes |
| 74 | TPX2 |  |  | 3006 | -0.606 | -0.0857 | Yes |
| 75 | DYNLL2 |  |  | 3036 | -0.625 | -0.0795 | Yes |
| 76 | DLG1 |  |  | 3043 | -0.628 | -0.0664 | Yes |
| 77 | SAC3D1 |  |  | 3073 | -0.655 | -0.0595 | Yes |
| 78 | LMNB1 |  |  | 3121 | -0.692 | -0.0569 | Yes |
| 79 | SMC4 |  |  | 3126 | -0.699 | -0.0416 | Yes |
| 80 | CCDC88A |  |  | 3138 | -0.714 | -0.0280 | Yes |
| 81 | KIF15 |  |  | 3142 | -0.723 | -0.0118 | Yes |
| 82 | CDK1 |  |  | 3149 | -0.727 | 0.0035 | Yes |
| 83 | MID1 |  |  | 3207 | -0.792 | 0.0056 | Yes |
| 84 | BIRC5 |  |  | 3208 | -0.793 | 0.0242 | Yes |
| 85 | FBXO5 |  |  | 3212 | -0.797 | 0.0421 | Yes |
| 86 | NUSAP1 |  |  | 3237 | -0.825 | 0.0545 | Yes |
| 87 | FGD4 |  |  | 3360 | -1.069 | 0.0441 | Yes |
Table: GSEA details [plain text format]

  

Fig 2: HALLMARK\_MITOTIC\_SPINDLE: Random ES distribution      
 Gene set null distribution of ES for **HALLMARK\_MITOTIC\_SPINDLE**
